# Accurate and efficient insulator maintenance: A DETR algorithm for drone imagery

**DOI:** 10.1371/journal.pone.0318225

**Published:** 2025-02-25

**Authors:** Yanfeng Tian, Rodina Binti Ahmad, Nor Aniza Binti Abdullah

**Affiliations:** Faculty of Computer Science and Information Technology, Universiti Malaya, Kuala Lumpur, Malaysia; King Fahd University of Petroleum & Minerals, SAUDI ARABIA

## Abstract

With the increasing demand for electricity, the safety and stability of power grids become paramount, highlighting the critical need for effective maintenance and inspection. Insulators, integral to power grid maintenance as protective devices on outdoor high-altitude conductors, are often subject to suboptimal image quality during drone-based inspections due to adverse weather conditions such as rain, snow, fog, and the challenges posed by sunlight, high-speed movement, and long-distance imaging. To address these challenges and achieve a more accurate inspection system, this manuscript introduces an insulator defect detection algorithm tailored for the low-quality images collected by drone-based imaging systems. Utilizing a patch diffusion model, high-quality images are obtained, enhancing the precision of insulator defect detection methods. Furthermore, to improve detection accuracy, we introduce an optimized DETR method that incorporates a Spatial Information Interaction Module to further strengthen the characteristics of minor defects. Additionally, a special convergence network is employed to augment the detection capabilities of the DETR. Experimental results demonstrate that our proposed insulator detection technique has achieved a detection accuracy of 95.8%, significantly outperforming existing defect detection methods in complex environments. It overcomes the drawbacks of traditional methods by employing sophisticated computational models, leading to more efficient, economical, and secure maintenance and inspection of power grids.

## 1. Introduction

In modern society, the demand for energy, especially electricity, continues to grow, driving the need for efficient and safe energy transmission systems. Building a modern energy system to promote efficient electricity use is a focal point in the development of power grids. Efficient electricity use largely depends on efficient power transmission, which highlights the importance of power grid maintenance and inspection. Insulators are essential components in the power system used for supporting and securing conductors while providing insulation, and are indispensable equipment in the power system. The failure of insulators can be catastrophic to the power grid. Therefore, it is crucial to detect insulator faults in a timely and accurate manner.

Insulators are critical electrical components in overhead power transmission systems. Their primary function is to secure and support cables while ensuring electrical isolation between cables and between cables and the ground. Insulators are often exposed to harsh environments for extended periods, leading to damage for various reasons. In dusty conditions, insulator surfaces may accumulate dirt and salts, which can become conductive when moist, thus reducing their insulating performance. Extreme weather conditions, such as hail, storms, strong winds, and high temperatures, can cause physical damage to insulators or affect their insulating capabilities. Additionally, over time, insulator materials, such as porcelain or glass, may age, weather, or crack, reducing their mechanical strength and insulating performance. These factors can lead to power grid failures. Therefore, developing methods for the rapid and accurate detection of insulator conditions is crucial for ensuring the stable operation of the power grid.

The insulator equipment that is difficult to inspect is mostly distributed in remote mountainous and forest areas [1]. Traditional detection methods include helicopters, climbing robots, and manual detection, which are costly and inefficient. With the development of drone technology and the goal of building intelligent power grids, drones have become the best way in power line inspections. However, drones’ detection faces a significant issue: the low-quality images captured by drones leads to low detection efficiency. This problem stems from various causes. Firstly, since drones need to capture raw image data from a distance away from power lines, it is inevitable that background noise will be introduced into the raw data. Secondly, drones may encounter air current disturbances during flight, causing the aircraft to vibrate. This vibration is transmitted to the camera, resulting in blurry images. Additionally, adverse weather conditions, such as strong winds, rain, or fog, can not only affect the stable flight of the drone but also lead to blurred or distorted aerial images. These are the objective reasons for the low quality of images collected by drones, which inevitably leads to a large number of low-quality images in the drones-acquired data, reducing the efficiency of drones’ detection. Therefore, noise reduction and image quality enhancement are key to improving the efficiency of drones’ detection.

Due to the complex structure and wide distribution of the power grid, insulator detection through drone images is an effective method to improve detection efficiency. However, in complex weather conditions, drones are unable to obtain high-quality images, increasing the difficulty of recognition. In light of this, there is an urgent need to develop a defect detection technology suitable for low-quality images captured by drones to improve the efficiency and accuracy of the detection work.

The solution proposed in this article consists of two parts. First, a patch diffusion model is used to generate high-quality images, reducing the impact of adverse weather conditions and lowering the difficulty of image recognition. Second, the optimized DETR model is employed to enhance the accuracy of small target identification. This comprehensive approach helps address the challenges posed by harsh weather conditions in UAV (Unmanned Aerial Vehicle) inspections, enabling all-weather inspections and ensuring the stable operation of high-voltage transmission lines.

Our work focuses on enhancing the efficiency and safety of power system electricity transmission, especially the detection efficiency of UAVs for insulators on power transmission lines under adverse weather conditions. We propose a method based on a patch diffusion model to generate high-quality images. This method can reduce the impact of adverse weather conditions on image quality and decrease the difficulty of image recognition. We employ an optimized DETR model to enhance the accuracy of small target identification, which is crucial for accurate detection by UAVs under complex weather conditions. Through the comprehensive application of the aforementioned technologies, the solution can tackle the weather challenges encountered during UAV inspections, achieving all-weather detection and ensuring the stable operation of high-voltage transmission lines. Considering the complexity and widespread distribution of the power grid structure, the solution is particularly suitable for insulator detection through UAV imagery, effectively improving detection efficiency, especially under adverse weather conditions, providing strong support for the safe and efficient operation of the power system.

## 2. Related works

The scheme adopted in this study for insulator detection based on low-quality images obtained under adverse weather conditions is divided into two parts. Firstly, denoise the image to obtain a high-quality image. Next, precise small object detection can be achieved through the optimized DETR model. Therefore, the related work is divided into two parts: image denoising techniques and object detection models.

### 2.1. Image denoising

Image denoising algorithms [2] are common noise reduction methods that can reduce random noise in images, such as Gaussian and median filtering. These algorithms are usually simple to compute, easy to implement, and deploy. However, while removing noise, they may blur the details of the image, which is disadvantageous for detecting small defects in insulators. Image enhancement techniques [3] can improve image quality through contrast enhancement, sharpening, and color balance adjustments, but they are mainly used to improve visual effects with limited denoising effects. Deep learning models, especially Generative Adversarial Networks (GAN), have been successfully applied to image denoising. However, they have high computational costs, and the training and inference phases may require significant computational resources. Under complex weather conditions, images captured by drones may contain rain and fog noise. To ensure the safety of high-voltage transmission lines, it is necessary to quickly and accurately detect insulator defects, which poses challenges.

Image denoising methods can be broadly categorized into two types: physics-based methods and deep learning-based methods. Physics-based methods rely on the atmospheric scattering model [4] and manually crafted priors, such as Dark Channel Prior, Color-line Prior [5], Color Attenuation Prior [6], Sparse Gradient Prior [7], Maximum Reflectance Prior [8], and Non-Local Priorr [9].

These manually crafted prior methods are primarily based on empirical observations. For example, Dark Channel Prior was proposed by Kaiming He et al. in 2009 [10], and its core principle is based on a key observation in natural images: in most non-sky local areas, some pixels in at least one color channel (red, green, or blue) have very low intensity values, close to zero, a phenomenon known as the “dark channel.” In the presence of haze, the haze increases the overall brightness of the image, making the intensity values of the dark channel higher. Therefore, by analyzing the intensity values of the dark channel, the concentration of haze can be estimated, and a haze-free image can be recovered. However, its effectiveness might be reduced when processing images of the sky or other areas without dark pixels, as the intensity values of the dark channel in these areas might not be low, leading to suboptimal denoising results. Additionally, the dark channel prior might cause the loss of some details in the image during the denoising process.

Moreover, these prior methods often have high computational costs. For instance, the Non-Local Prior requires considering a large amount of non-local information in the image, making the algorithm implementation relatively complex, optimization and parameter tuning difficult, and the computational cost extremely high. More importantly, these prior methods might not recover all details and could even over-enhance certain areas during the denoising process, leading to detail loss. This is particularly detrimental for tasks such as detecting defects in insulators.

In recent years, deep learning methods have made progress in image denoising, with diffusion models [11], in particular, attracting increasing attention due to their ability to generate high-quality images through an iterative optimization process. Diffusion models can transform hazy images into clear ones, possessing desirable attributes such as distribution coverage, stable training objectives, and scalability [12]. Following this direction, DDPM (Denoising Diffusion Probabilistic Model) [13] have been developed for low-level vision image enhancement tasks, such as image super-resolution [14], image restoration [15], and image deblurring [16]. Although DDPM-based methods have been developed for some low-level vision tasks, their use in image denoising is unprecedented.

However, DDPMs have not accounted for the physical properties of the denoising task, limiting their ability to complete information in hazy images. This article introduces Conditional DDPM to address the challenging task of denoising images in dense fog. Conditional DDPM is a diffusion model capable of incorporating conditional information. It extends the traditional DDPM, allowing the model to consider additional conditional information during the generation process [17]. By incorporating conditional information (such as a degraded image $x_{d}$) into the reverse process, these models are able to more precisely control the generation process, thus producing more predictable and high-quality results. Conditional diffusion models permit the adjustment of the intensity of conditional information during the generation process, thereby controlling the typicality and diversity of the generated samples. By tuning the conditional weight parameter, a smooth transition can be made between unconditional and conditional generation [18]. Conditional DDPM enhances the model’s generative capabilities by integrating conditional information.

### 2.2. Object detection model

In terms of detection methods, Convolutional Neural Networks (CNNs) have shown immense potential in remote sensing image object detection models. CNNs can automatically learn multi-scale features of images through their multi-layered structure. For instance, the Feature Pyramid Network (FPN) [19] can effectively combine features from different levels, thereby enhancing the accuracy and robustness of detection. Models based on Faster R-CNN [20] have significantly improved detection speed and accuracy by introducing the Region Proposal Network (RPN) [21], making them more suitable for processing large-scale remote sensing datasets. U-Net [22] employs an end-to-end Fully Convolutional Network (FCN) [23] for semantic segmentation of remote sensing images, enabling the identification and classification of objects within images. However, the detection of small objects in remote sensing images remains a challenge for CNNs, as these objects occupy few pixels in the image and may be obscured by each other. This can lead to poor detection performance, especially in scenarios requiring precise localization of small objects. This means that CNNs may need to be combined with other types of networks to better handle small object detection tasks.

DETR (Detection Transformer) [24] is an object detection model based on the Transformer architecture, which transforms the object detection problem into a direct set prediction problem. DETR is an end-to-end object detection model that directly predicts the bounding boxes of targets in images through a Transformer encoder-decoder structure, meaning it can learn the entire process from images to target detection results in one go, without the need for additional region proposal networks or post-processing steps. Due to the characteristics of the Transformer architecture, DETR is able to capture global contextual information, which is very important for understanding image content and improving detection performance. DETR uses self-attention mechanisms to process image features, enabling the model to better understand and utilize long-range dependencies in images.

However, although the DETR model introduces new ideas and methods in the field of object detection, it also has some limitations and shortcomings. Compared to some traditional object detection models, DETR converges more slowly and requires more training epochs to achieve good performance. The training process of DETR is relatively complex, requiring special training strategies, such as Hungarian matching and special loss functions, which increases the complexity of training. Additionally, DETR performs poorly in detecting small object, especially when objects are small in size or partially occluded. To address these issues, researchers are exploring various improvement strategies, such as model architecture improvements, training strategy optimizations, and loss function adjustments, to enhance the performance and applicability of DETR. With the continuous development of deep learning technology, it is expected that more innovative methods will emerge in the future to overcome these challenges. Zhu et al. employed a deformable self-attention mechanism [25], strategically sampling key regions of the image and integrating multi-scale features to enhance the recognition of small objects. Furthermore, Dai et al. developed dynamic attention techniques [26], dynamically adjusting based on the importance of scale, spatial positioning, and feature dimensions to optimize performance and accelerate model convergence.

These studies have provided broad ideas and achieved results for optimizing DETR models. This study focuses on the task of insulator image detection, improves the model architecture and adjusts the loss function, and proposes an optimized model based on DETR to improve the performance of detecting small objects. Its simplified workflow and excellent performance have broad application prospects in the field of imaging.

## 3. Solution for low-quality UAV image detection

### 3.1. Overview

**Fig 1:**
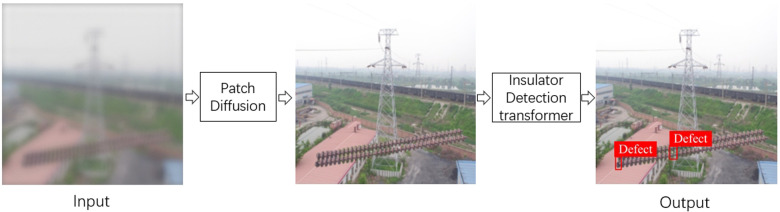
IDD-DETR model structure diagram.

Current research has reached a high accuracy in the detection of insulator defects. However, most research focuses on fault detection under normal weather conditions and does not pay sufficient attention to adverse weather conditions. To address the challenge of insulator defect identification for the low-quality images captured by drones, we propose the Insulator Defect Detection DETR (IDD-DETR) model based on the patch diffusion model, as shown in Fig 1.

**Fig 2:**
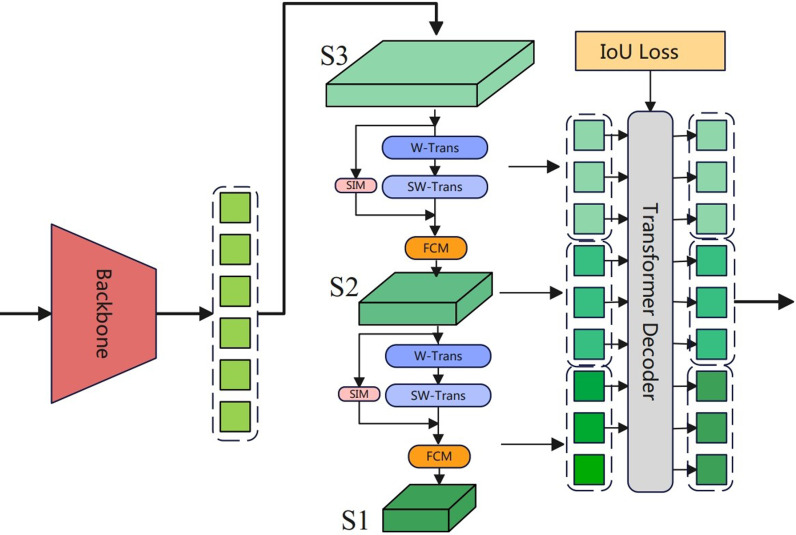
Structure of insulator detection transformer.

Through the diffusion model, we obtain high-quality images to enhance the accuracy of insulator defect detection methods. Moreover, to further improve detection accuracy, we introduce an insulator detection transformer approach, which incorporates a Spatial Information Interaction Module (SIIM) to further strengthen the features of small defects, as shown in Fig 2. Additionally, by using a Feature Convergence Module (FCM), we integrate the global information of the Transformer into CNN features to improve recognition accuracy, thereby enhancing the detection performance of DETR.

### 3.2. Patched based diffusion image restoration

Diffusion models have been widely used in image generation and restoration in recent years. The core idea behind diffusion models is to progressively introduce noise into the original data until it becomes pure noise, and then use a learned reverse process to remove the noise step by step, transforming it back into clear data. The advantage of diffusion models lies in their ability to generate high-quality images, but traditional unconditional diffusion models often result in uncontrollable and random outcomes.

To address this issue, conditional diffusion models have emerged. By incorporating conditional information (such as a degraded image $x_{d}$) into the reverse process, these models are able to more precisely control the generation process, thus producing more predictable and high-quality results. The core objective of conditional diffusion models is to learn a conditional reverse process $p_{\theta }\left(x_{0:T}\text{|}x_{d}\right),$ without altering the diffusion process $q\left(x_{1:T}\text{|}x_{0}\right).$ In training, the model learns how to incorporate the degraded image $x_{d}$ as a condition, guiding the reverse process to ensure that the generated images are more faithful to the data distribution conditioned on $x_{d}.$

During the training of a denoising model, data pairs consisting of clear images $x_{0}$ and degraded images $x_{d}$ are constructed. The model samples from these data pairs, learning a conditional diffusion model that takes the degraded image $x_{d}$ as input during the reverse process:


$$p_{\theta }\left(x_{0:T}\text{|}x_{d}\right)=p\left(x_{T}\right)\prod_{t=1}^{T}p_{\theta }\left(x_{t-1}\text{|}x_{t},x_{d}\right)$$ (1)

#### 3.2.1. Patch decomposition and optimization.

The key idea of patched-based diffusion models is to decompose the image into smaller patches and then model each patch independently. This approach allows the model to better capture local image features and improve the quality of restoration by focusing on local details.

Assume we have a ground truth image $x_{0}$ (of arbitrary size) and a degraded image $x_{d}$. These images are decomposed into multiple p×p patches. Each patch is processed independently, and the restoration of each patch is optimized. To efficiently model these patches, a binary mask matrix $p_{i}$ is introduced, which indicates the position of the i-th patch in the image.

#### 3.2.2. Conditioned patch reverse process.

For image restoration using patched-based diffusion, a conditional reverse process is learned, but this process is applied independently to each patch. During training, we use pairs of ground truth patches $x_{0}^{i}$ and degraded patches $x_{d}^{i}$ as training data. The objective is to learn the conditional reverse process for each patch, as represented by:


$$p_{\theta }\left(x_{0:T}^{i}\text{|}x_{d}^{i}\right)=p\left(x_{T}^{i}\right)\prod_{t=1}^{T}p_{\theta }\left(x_{t-1}^{i}\text{|}x_{t-1}^{i},x_{d}^{i}\right)$$ (2)

$x_{0}^{i}$ and $x_{d}^{i}$ denoting p ×  p patch from training set image pair. During training, random sample of the p ×  p patch location for $p_{i}$ within the image are generated.

#### 3.2.3. Patch-level training and sampling.

1. Data pair construction: Extract the clear image $x_{0}$ and the degraded image $x_{d}$ from the image pairs, and decompose them into p×p patches.
2. Patch-level training: Model the generation process of each patch independently through the aforementioned conditional diffusion model (Equation 2). During training, the position and content of each patch are randomly selected, and the model is optimized based on the local information of the patches.
3. Reverse process sampling: In the testing phase, the model performs reverse sampling based on the patch information in the degraded image $x_{d}$, gradually restoring the details of each patch, and finally merging the restored patches to obtain the final restored image.

By employing this method, the patch-based diffusion model can effectively utilize local information in image restoration tasks, improving the quality of restoration and reducing errors caused by noise in the overall generation process.

#### 3.2.4. Advantages.

- Local feature optimization

By individually modeling each patch of the image, the approach can more finely capture local features within the image.

- Higher restoration accuracy

The patch-based processing allows the model to effectively handle details in the image and reduces distortion when merging patches.

- Flexible control

The model can flexibly optimize each patch, thereby more accurately restoring damaged parts.

In summary, the patch-based diffusion model optimizes the image restoration process through the handling of local patches, enabling image generation and restoration tasks to enhance restoration quality and efficiency while ensuring the preservation of details.

### 3.3. Insulator defect detection transformer

#### 3.3.1. Spatial Information Interaction Module (SIIM).

**Fig 3:**
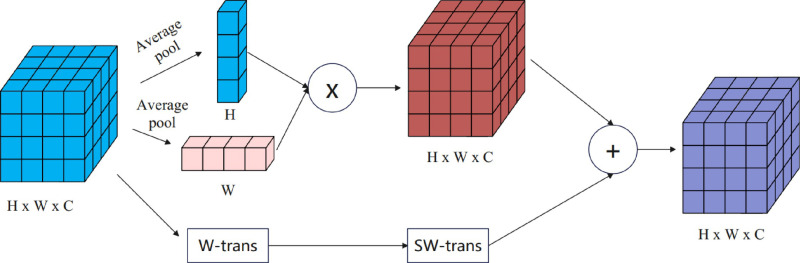
The structure of the SIIM module.

In the IDD-DETR model, the use of Swin Transformer [27] effectively reduces memory overhead and establishes relationships between patch tokens within a limited window. However, this approach somewhat diminishes the global modeling capability, even with the implementation of an alternating strategy between regular and shifting windows. Moreover, in remote sensing images, object occlusion leads to blurred boundaries, necessitating the elimination of certain spatial information. Therefore, we introduce the Spatial Information Interaction Module (SIIM) after SW-Trans to further enhance information interaction while encoding more precise spatial details. SIIM introduces attention in two spatial dimensions, considering the relationships between individual pixels, not just limited to the relationships between patch tokens, thereby enabling the Transformer to exhibit outstanding performance in image detection tasks. The composition of SIIM is depicted in Fig 3. By integrating the SIIM module into the Swin Transformer, our model demonstrates significant performance improvements in remote sensing image segmentation tasks.

#### 3.3.2. Feature Convergence Module (FCM).

**Fig 4:**
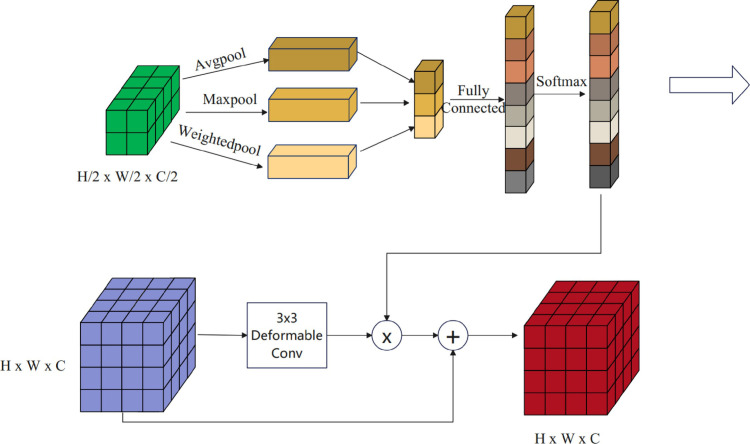
The structure of the FCM module.

The main encoder based on CNN technology can effectively extract local information restricted by the convolution kernels in the spatial dimension, but it lacks explicit modeling of the relationships among the whole. To address this issue, we designed a feature fusion module, as shown in Fig 4.

The feature fusion module acts as a bridge between the dual encoders, hierarchically integrating the global dependencies of the Swin Transformer into the CNN features, achieving the fusion of features from the two branches. One branch utilizes the ResNet [28] backbone network, while the other branch encoder employs the Swin Transformer. By introducing the RAM module, we integrate the global information of the Swin Transformer into the CNN features, thereby better capturing the overall interrelations and enhancing the model’s performance and representation.

## 4. Experiment

### 4.1. Data preparation

This study used the following publicly available datasets.

- CPLID dataset

The Chinese Power Line Insulator Dataset (CPLID) is specifically designed for the research and development of automatic detection and diagnostic technologies for high-voltage power line insulators. This dataset contains a vast array of high-voltage line insulator images, aimed at aiding researchers and developers in utilizing machine learning and computer vision technologies to enhance the accuracy and efficiency of insulator defect detection. The CPLID dataset typically includes three categories of images. The first category consists of normal insulators, which display insulators without any defects or damage. The second category includes damaged insulators, featuring images of insulators with various common types of damage such as cracks, breakages, contamination, and flashover marks. The CPLID dataset also contains images of insulators under different environmental conditions. To simulate a variety of real-world environmental conditions, the dataset may include images taken under different weather conditions (such as sunny, rainy, or foggy) and at different times (such as day and night).

The images in the CPLID dataset usually come from high-voltage transmission lines across different regions, covering a variety of types and backgrounds of insulators to enhance the generalizability of models. All images in the CPLID dataset are accompanied by detailed annotations, including the type of insulator, type of damage, and the specific location of the damage, which assists in training more precise detection models.

- UPID dataset

Unifying Public Datasets for Insulator (UPID) was constructed by augmenting the CPLID insulator image data. Albumentations API was utilized to apply various methods like Gaussian blur and random affine transformation on the original CPLID dataset, Experiment Process and Analysis resulting in a new dataset that contains a total of 6860 insulator images.

- SFID dataset

Synthetic Foggy Insulator Dataset (SFID) contains images of insulators generated in synthetic foggy weather. This dataset was created by enhancing the UPID dataset through methods such as applying random brightness and adjusting fog thickness. The dataset contains a total of 13718 insulator images.

In the field of image restoration, synthetic data is often used as data source, rather than real adverse weather images, as it is challenging to obtain images of the same detection target in both normal and adverse weather conditions. To address this, we obtained data from the CPLID dataset and divided it into training and testing sets. Using DDRM [16], we generate synthetic insulator images with two types of degradation: Gaussian blur and a uniform blurring method from DDRM. We selected insulator image data from the degraded CPLID dataset to train a diffusion model that specifically handles foggy insulators and the original image data from CPLID to fine-tune the model.

### 4.2. Training settings

In the image decontamination stage, we utilized the model training method under the guidance of the denoising algorithm proposed by Weather Diffusion [29] to improve the denoising effect of the original model. The Weather Diffusion pre-trained model is a patched-based conditional diffusion model, and was trained with three adverse weather datasets from the outdoor environment, for the purpose of desnowing, deraining, dehazing, and raindrop removal. Although this model exhibited high performance in an outdoor environment with adverse weather, it showed poor results with denoising foggy insulation images in outdoor scenarios.

In this study, our training environment was built on a high-performance computing node, and the detailed configuration is as in Table 1:

**Table 1:** Experimental environment configuration.

Item	Detail
CPU	Dual Intel Xeon Gold 6248R (48 physical cores, 96 threads)
RAM	512 G DDR4 memory
GPU	1 NVIDIA A100 GPU with 40GB of graphics memory
Storage	NVMe solid-state drive for fast data loading and intermediate result writing
Operating System and Drivers	Running in Linux environment, GPU driver version meets CUDA 11.8 and cuDNN corresponding dependencies
Framework and dependencies	PyTorch version 1.8 and its related dependencies to accelerate model training and inference.

The training process set the batch size to 8 and trained for 50,000 iterations with an Adam optimizer and a learning rate of 0.0002, while the weight decay was set to 0. The training process adopted a patch-based denoising diffusion model.

The training of DETR generally requires a larger number of training iterations for the model to converge fully. Related research indicates that the number of iterations needed to stabilize model performance typically ranges from tens of thousands to hundreds of thousands, depending on the complexity of the training dataset. 50,000 iterations represent a balanced figure between reasonable training resources (GPU time) and the enhancement of model performance. In preliminary experiments, we observed the change curves of the validation set loss and mAP metrics with the number of iterations. When training reaches around 30,000 to 40,000 iterations, the mAP metric essentially stabilizes, and further increasing to 50,000 iterations allows the model to reach a better stable point. Continuing to extend training time beyond this point (such as 60,000 or 70,000 iterations) shows diminishing marginal returns in performance improvement. Under the hardware configuration of Table 1, 50,000 iterations keep the overall training duration within an acceptable range (within a few days), which can meet research progress requirements while ensuring the model has ample opportunities to learn.

Due to the inclusion of the Transformer module in the DETR model, it is quite sensitive to the learning rate. An excessively high learning rate may lead to gradient explosion or unstable convergence, while a too low learning rate can cause slow training progress. Through multiple attempts between 1e-4 and 5e-4, we found that 0.0002 can quickly reduce the loss in the initial stage of training and achieve a more robust convergence performance. At the same time, the original paper of DETR and subsequent studies often set the initial learning rate within a similar range, providing a reference for the choice of 0.0002.

Our training process accepts normal and foggy images as pair, a patch sample will be generated to evaluate the denoising effect every 10,000 iterations. The training process utilized 480 normal insulator images and 198 defective insulator images that were degraded using the DDRM hazing degradation algorithm [16].

### 4.3. Compared with other methods

In order to evaluate the effectiveness of denoising, a comparison was made between the trained Weather Diffusion model, the original Weather Diffusion model, and the DDRM model, since the original Weather Diffusion model and DDRM were not intentionally trained for the denoising of foggy insulator images. The test set contained 120 images of normal insulators and 50 images of defective insulators, randomly chosen from the original CPLID dataset which were not used in the model training process. The degraded images of the insulators were generated by using a degrader provided by the DDRM model with two types of blur degradation. Comparisons have been conducted by performing direct comparisons and using conventional peak signal-to-noise ratio (PSNR) and structural similarity (SSIM) metrics to conduct quantitative evaluations between ground-truth images and restored images. PSNR and SSIM metrics indicate the evaluation between the restored images and ground-truth images, the higher the value, the smaller the loss, and the image distortion, respectively.

**Fig 5:**
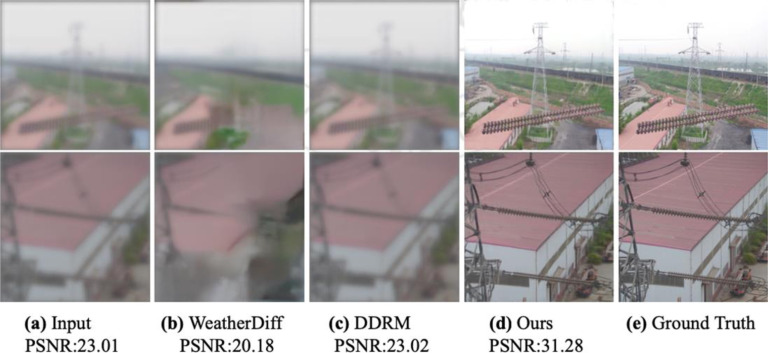
Comparison of three models.

Fig 5 shows the comparison between three models in the visual aspect. Input figures are degraded by uniform blur and Gaussian blur from the top to the bottom, and image data from the defective insulator and normal insulator, respectively. The result shows that Weather Diffusion and DDRM could not dehaze properly, while Weather Diffusion loses part of the information of the insulator and halved the size of the insulator during image generation. DDRM appears making the shape of insulator clearer, however leaving too much haze on the generated image. Our trained model generates clearer images and restores more detail of the insulator, which is crucial to insulator fault detection, although it still loses some detail compared to ground-truth images. Table 2 shows that our model performs well on both PSNR and SSIM metrics.

On the SFID dataset, we conducted a series of comparative analyses, pitting our developed IDD-DETR model against several advanced DETR variants as well as traditional CNN-based object detection methods. From the data presented in Table 3, it is evident that although IDD-DETR has fewer model parameters, it surpasses the other comparison models in both precision and recall, two critical performance metrics. This indicates that IDD-DETR maintains a lightweight model while not compromising on performance; instead, it demonstrates enhanced detection capabilities. Moreover, it is particularly noteworthy that IDD-DETR has shown significant progress in detecting small defects (APs). This achievement is crucial because detecting small defects is often more challenging, requiring the model to have higher sensitivity and more refined feature extraction capabilities. Our IDD-DETR, through optimized attention mechanisms and feature fusion strategies, effectively improves the model’s accuracy in recognizing small-sized targets.

**Table 3:** Comparison of different methods in SFID.

Model	Backbone	#Epcohs	#Param (M)	FLOPS	Precision (%)	Recall (%)	F1 Score	Aps (%)
Faster-RCNN [21]	R50	24	–	–	80.5	65.2	72.0	29.5
ATSS [30]	R50	24	–	–	80.6	70.3	75.1	32.5
YOLO v5 [31]	–	275	–	–	81.3	67.7	73.9	28.5
YOLO v7 [32]	–	275	–	–	89.5	86.7	88.1	30.2
IYOLO v7 [33]	–	275	–	–	91.1	88.4	89.7	34.3
Deformable-DETR [34]	R50	50	40.2	170.1	81.2	81.5	81.3	26.2
Conditional-DETR [35]	R50	50	43.3	63.7	82.1	68.3	74.6	25.5
Group-DETR [36]	R50	50	44.1	63.2	78.3	78.2	78.2	31.6
DINO [37]	R50	12	47.6	178.4	89.8	83.4	86.5	33.7
RT-DETR [38]	R50	72	41.4	93.4	90.6	87.5	89.0	34.1
IDD-DETR (Ours)	R50	72	39.3	90.1	95.8	90.6	93.1	36.3

Note: The missing part of the data is based on CNN methods, which are not comparable to those based on DETR methods.

The YOLO (You Only Look Once) series models are real-time object detection models based on CNN. YOLO v7 and IYOLO v7 are representatives of high performance among them. Compared with the average precision of the CNN based model in the experiment, our IDD-DETR model is 11.2% higher.

Deformable-DETR, Conditional-DETR, Group-DETR and RT-DETR are currently popular object detection models, both of which are enhanced and improved models based on DETR. DINO is a model based on anomaly detection. Compared to the best performing RT-DETR model among them, our IDD-DETR model has a 5.2% higher precision.

**Fig 6:**
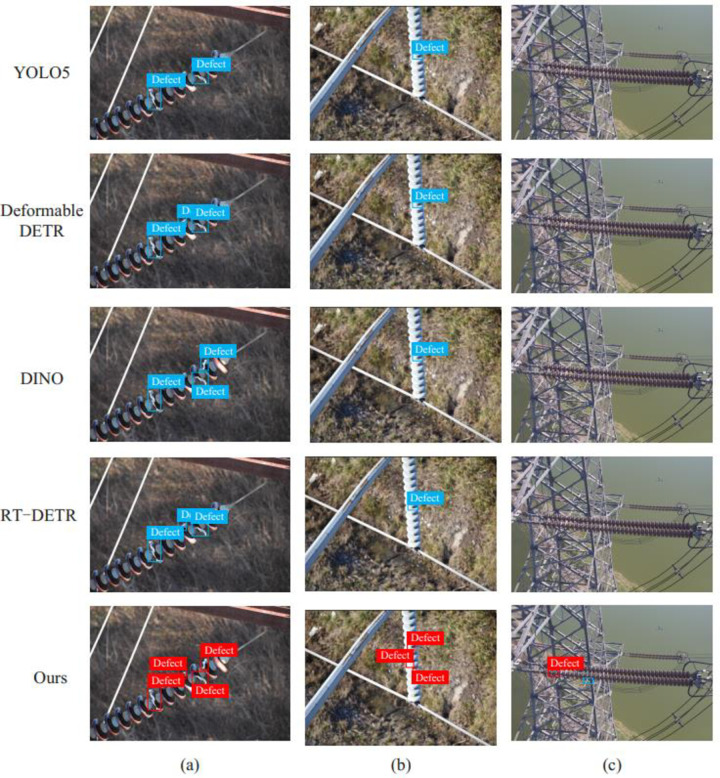
The detection results of different methods.

Fig 6 illustrates the detection outcomes from four advanced methods alongside our own. The results reveal that while Deformable-DETR and RT-DETR struggle with the detection of small defects, leading to missed detections especially noted in column b of Fig 6, our algorithm excels. It accurately identifies small craters, which have less pronounced edge features, showcasing superior detection abilities for such objects.

In column c of Fig 6, our model demonstrates a clear advantage in processing images with intricate backgrounds. The detailed texture patterns in these backgrounds, which can mimic defects, present a substantial detection challenge. Despite these complexities, our approach remains largely unaffected and successfully identifies defects that other methods overlook. This success is attributed to the innovative self-attention upsampling module in our model, which adeptly distinguishes between the foreground and background, thereby improving feature learning and enhancing the overall robustness of the model.

The results of the UPID dataset are presented in Table 4. The model trained with the foggy dataset achieved a comprehensive improvement in Precision, Recall, mean Average Precision (mAP), and F1 score compared to the model trained with the fog-free dataset. Performance on the fog-free test set was also evaluated, with an F1 score of 91.2%. Although this score is significantly lower than the 92.5% achieved on foggy test sets, it is still suitable for the application of insulator defect detection. This demonstrates that the data-augmented model not only shows improvement on foggy days but also exhibits an overall enhancement in performance.

**Table 4:** Results of the UPID dataset.

Dataset	number	Precision	Recall	mAP	F1
foggy	4318	88.6	96.1	96.3	92.5
fog-free	5078	87.5	95.3	95.6	91.2

Overall, our model demonstrates distinct strengths in feature learning and in differentiating foreground from background elements. It enhances the precision of detecting defects, particularly small ones, by employing advanced multi-scale feature fusion methods and a more reliable matching process.

**Table 2:** Comparison on PSNR and SSIM metrics.

	PSNR	SSIM
Weather Diffusion	20.18	0.52
DDRM	23.02	0.58
Patch Diffusion (Ours)	31.28	0.69

### 4.4. Ablation study

We conducted ablation studies to assess the contribution of each component to the model’s performance. The IDD-DETR model proposes two innovative modules based on RT-DETR. In the ablation experiments, the SIIM and FCM modules were gradually incorporated into the baseline model, and the model effects were observed, with the results shown in Table 5. First, we used the SIIM in the classic DETR, which improved the defect detection accuracy by 1.9%, highlighting the advantage of multi-scale feature fusion under the DETR framework. Subsequently, the FCM was integrated into the baseline model alone, and the model’s detection accuracy improved by 2.6%, reflecting the effectiveness of the FCM module in the detection of minor defect features. Finally, by using both the FCM and SIIM modules simultaneously and integrating the FCM into the multi-scale bottom-up pathway, the model’s accuracy increased by 5.2% over the baseline. In summary, our experiments demonstrate that each component of the proposed algorithm can effectively enhance the model’s performance, and they work in coordination with each other without conflicts.

**Table 5:** Influence of each component.

Model	SIIM	FCM	Precision (%)	Recall (%)
Baseline	–	–	90.6	87.5
Baseline + Parts	√	–	92.5 ↑ 1.9	88.3 ↑ 0.8
	–	√	93.2 ↑ 2.6	88.9 ↑ 1.4
	√	√	95.8 ↑ 5.2	89.6 ↑ 2.1

### 4.5. Generalization study

To further validate the broad applicability of the IDD-DETR model in insulator defect detection, we conducted experiments on the CPLID dataset. According to the results in Table 6, our model continues to demonstrate exceptional detection capabilities. Compared to the best RT-DETR model among other algorithms, our method outperforms in precision by 8.4%, and it is 11.14% more precise on average than other models. Compared to the experiments on the SFID dataset, the IDD-DETR model’s performance is even more prominent. This is because the CPLID dataset includes insulator images under different environmental conditions, such as images taken under various weather conditions (e.g., sunny, rainy, or foggy days) and at different times (e.g., day and night). Images of harsh environments pose challenges to the model. The defogging treatment of the IDD-DETR model showcases its ability to recognize low-quality images. This indicates that our method can effectively process low-quality images collected by drones and has strong robustness, achieving better experimental results under various data distributions. This is attributed to the series of innovations we proposed, which are more conducive to practical industrial applications.

**Table 6:** Comparison of different methods in CPLID dataset.

Model	Precision (%)	Recall (%)
YOLO-5 [31]	86.2	86.3
Deformable-DETR [34]	81.2	76.4
Focus-DETR [39]	83.5	81.2
DINO [37]	87.1	84.1
RT-DETR [38]	88.3	90.6
IDD-DETR (Ours)	96.4	93.7

## 5. Discussion

### 5.1. Contributions

This study focuses on exploring the application of DETR variants in insulator defect detection, particularly highlighting the enhanced capability for detecting minute defects. First, denoise the drone images to mitigate the impact of adverse weather conditions. After processing foggy images with Patch Diffusion, the PSNR value is higher than that of Weather Diffusion and DDRM models, reaching 31.28 DB. This is an ideal score; typically, a PSNR value above 30 dB indicates good image quality. Second, we conducted a series of comparative analyses, pitting our developed IDD-DETR model against several advanced DETR variants (such as RT-DETR) as well as traditional CNN-based object detection methods (such as IYOLO v7) On the SFID dataset. The proposed IDD-DETR model achieves a precision of 95.8% and a recall of 90.6%, outperforming RT-DETR (with precision of 90.6% and recall of 87.5%) and the best-performing CNN-based model, IYOLO v7 (with precision of 91.1% and recall of 88.4%). This demonstrates that IDD-DETR has certain advantages in the detection of insulator defects.

The advantages of DETR and its derivatives across multiple object detection tasks, yet several challenges persist in the specific context of insulator defect detection. On one hand, the reduction in feature layer sizes with the deepening of the network leads to the loss of features related to minute defects. On the other hand, the self-attention mechanism in the DETR model might overly amplify the information within these minute defects, causing confusion with background features.

To address these challenges, the research proposes an insulator defect identification model suitable for low-quality images captured by drones, initially generating high-quality images using a patch diffusion model. Subsequently, the SIIM is used to enhance information interaction, improving recognition precision with accurate spatial details. The FCM acts as a bridge between the dual encoders, integrating global in-formation from the Swin Transformer into CNN features to enhance recognition ac-curacy. Experiments on multiple public datasets have demonstrated that IDD-DETR offers higher accuracy and efficiency in insulator defect detection for low-quality images captured by drones.

### 5.2. Limitations and future research directions

While the research has made progress, there are also some limitations. For example, the datasets currently used may not comprehensively cover all types of insulator defects, especially those such as internal cracks that are difficult to detect through external images. Moreover, although the model performs well on selected datasets, its generalization ability across different grid structures and environmental conditions has yet to be validated. The efficiency of defect detection under more complex backgrounds and the computational efficiency and processing speed of the model in real-time or near-real-time applications are also issues that need to be focused on in the future.

In the face of these challenges, future research directions include: expanding and diversifying the datasets to enhance the model’s generalization ability; further investigating the model’s adaptability under different grid structures and environmental conditions; exploring algorithm optimization and model architecture innovation to improve computational efficiency; developing advanced background processing techniques to improve detection accuracy in complex environments; and integrating multi-source data and utilizing multimodal learning approaches to further enhance the accuracy and reliability of insulator defect detection.

## 6. Conclusion

This study made significant contributions to the power transmission industry by addressing the challenge of insulator fault detection under adverse weather conditions, specifically in foggy environments. Insulators play a crucial role in the power transmission industry. Insulator fault detection based on deep learning methods is mainly conducted under normal weather conditions, with less attention given to insulators under adverse weather conditions. This paper proposes a method that combines the optimized DETR and the patch diffusion model, achieving denoising of foggy insulator images and fault detection, ultimately aiming for insulator fault detection for low-quality images captured by drones. Experimental results show that, compared to the original model, the proposed model achieves better performance in both foggy insulator image restoration and faulty insulator image detection after training and fine-tuning. This proves that the objective of detecting insulator faults under adverse weather conditions has been achieved.

Future research should focus on enhancing the fault detection capabilities of the IDD-DETR model. The current method is limited to detecting visible defects without identifying the underlying causes, such as explosions or missing covers, due to the lack of comprehensive labeling in available datasets. To advance, we recommend the development of more detailed and extensive datasets that can provide the necessary depth for the model to understand and predict a wider range of insulator defects. This will involve not only collecting more data but also enriching the annotations to include the root causes of the defects. The ultimate aim is to refine the IDD-DETR model to a point where it can predict and prevent insulator failures before they occur, enhancing the overall reliability and safety of power transmission systems. In addition, we will explore the application of IDD-DETR models in other small object detection fields, such as medical imaging and industrial production.
